# Microglial Morphology Across Distantly Related Species: Phylogenetic, Environmental and Age Influences on Microglia Reactivity and Surveillance States

**DOI:** 10.3389/fimmu.2021.683026

**Published:** 2021-06-18

**Authors:** Dario Carvalho-Paulo, João Bento Torres Neto, Carlos Santos Filho, Thais Cristina Galdino de Oliveira, Aline Andrade de Sousa, Renata Rodrigues dos Reis, Zaire Alves dos Santos, Camila Mendes de Lima, Marcus Augusto de Oliveira, Nivin Mazen Said, Sinara Franco Freitas, Marcia Consentino Kronka Sosthenes, Giovanni Freitas Gomes, Ediely Pereira Henrique, Patrick Douglas Côrrea Pereira, Lucas Silva de Siqueira, Mauro André Damasceno de Melo, Cristovam Guerreiro Diniz, Nara Gyzely de Morais Magalhães, José Antonio Picanço Diniz, Pedro Fernando da Costa Vasconcelos, Daniel Guerreiro Diniz, Daniel Clive Anthony, David Francis Sherry, Dora Brites, Cristovam Wanderley Picanço Diniz

**Affiliations:** ^1^ Laboratório de Investigações em Neurodegeneração e Infecção, Instituto de Ciências Biológicas, Hospital Universitário João de Barros Barreto, Universidade Federal do Pará, Belém, Brazil; ^2^ Faculdade de Fisioterapia e Terapia Ocupacional, Universidade Federal do Pará, Belém, Brazil; ^3^ Laboratório de Biologia Molecular e Neuroecologia, Instituto Federal de Educação Ciência e Tecnologia do Pará, Bragança, Brazil; ^4^ Laboratório de Microscopia Eletrônica, Instituto Evandro Chagas, Belém, Brazil; ^5^ Dep. de Arbovirologia e Febres Hemorrágicas, Instituto Evandro Chagas, Belém, Brazil; ^6^ Departamento de Patologia, Universidade do Estado do Pará, Belém, Brazil; ^7^ Department of Pharmacology, University of Oxford, Oxford, United Kingdom; ^8^ Department of Psychology, Advanced Facility for Avian Research, University of Western Ontario, London, ON, Canada; ^9^ Research Institute for Medicines (iMed.ULisboa), Faculty of Pharmacy, Universidade de Lisboa, Lisbon, Portugal; ^10^ Department of Pharmaceutical Sciences and Medicines, Faculty of Pharmacy, Universidade de Lisboa, Lisbon, Portugal

**Keywords:** age and environment influence on microglia alteration, brain size and microglia response cognitive performances, Felsenstein’s independent phylogenetic contrast, microglia, mouse, bat, semipalmated sandpiper microglia morphology

## Abstract

Microglial immunosurveillance of the brain parenchyma to detect local perturbations in homeostasis, in all species, results in the adoption of a spectrum of morphological changes that reflect functional adaptations. Here, we review the contribution of these changes in microglia morphology in distantly related species, in homeostatic and non-homeostatic conditions, with three principal goals (1): to review the phylogenetic influences on the morphological diversity of microglia during homeostasis (2); to explore the impact of homeostatic perturbations (Dengue virus challenge) in distantly related species (*Mus musculus* and *Callithrix penicillata*) as a proxy for the differential immune response in small and large brains; and (3) to examine the influences of environmental enrichment and aging on the plasticity of the microglial morphological response following an immunological challenge (neurotropic arbovirus infection). Our findings reveal that the differences in microglia morphology across distantly related species under homeostatic condition cannot be attributed to the phylogenetic origin of the species. However, large and small brains, under similar non-homeostatic conditions, display differential microglial morphological responses, and we argue that age and environment interact to affect the microglia morphology after an immunological challenge; in particular, mice living in an enriched environment exhibit a more efficient immune response to the virus resulting in earlier removal of the virus and earlier return to the homeostatic morphological phenotype of microglia than it is observed in sedentary mice.

## Introduction

The processes of microglia extend and retract continuously, guided by its sensome (1), to survey their non-overlapping territorial limits. Microglia represent the most important cellular component of the innate immune response in the central nervous system (2–4). However, the number of possible homeostatic functions of microglia in the healthy central nervus system (CNS) remains poorly defined and the matter of much speculations (4, 5). Microglia show regional and heterogeneous morphological features in the CNS, with large variability on their processes, cell size and gene expression, across species (6). Although microglia constitute approximately 7% of non-neuronal cells in different brain structures, as well as in the whole brain of all mammalian species examined to date (7), their diversified phenotype and extended contributions to maintain CNS homeostasis is considerable (8). Under homeostatic conditions, microglial ramifications survey the surrounding environment, monitor the synapses’ functional state, regulate neuronal activity in all phases of development and in adult life, and participate in the remodeling and maturation of synaptic circuits, through their contacts with the pre- and post-synaptic regions (9–11); for a recent review, see (12). While scanning the brain parenchyma to detect local changes, microglia show a myriad of morphological and functional changes with considerable overlap (3, 13, 14). As a function of environmental stimuli, age or nature of the imposed insult, microglia may differentially respond to the threats (15, 16), establishing new relationships between form and function (13, 14, 17–19). Today, it is also known that extrinsic and intrinsic key factors, including host microbiota, influence microglia functions and states (20) and that microRNAs, a class of small (~22 nucleotides) single‐stranded noncoding ribonucleic acid (RNAs), are drivers of microglia phenotypic changes (21). Evidence also demonstrate that there are multiple reactive microglial states that are selected by the discrimination of discrete perturbations within the brain parenchyma, in both physiological and pathological conditions (22); each state of activation is selected to make its own distinct physiological contributions, regulated by purinergic mechanisms, to changes in the local environment.

To search for morphological differences across phylogenetically distantly related species we reexamined our previous morphometric findings based on microglia three-dimensional reconstructions and compared data obtained in the dentate gyrus of mouse (*Mus musculus*) (23) and hippocampus V area of the semipalmated sandpiper (*Calidris pusilla*) (24), with those of microglia of the dentate gyrus of the lesser bulldog bat (*Noctilio albiventris*, unpublished results). The species selected allow the comparison of similar body weights and brain volumes for the microglia morphological categorization in the dentate gyrus, associated to contrasting locomotor and cognitive performances and distinct phylogenetic histories. Using phylogenetic trees generated by nuclear recombination-activating gene (RAG-1) and mitochondrial cytochrome oxidase (COI) markers of these three species, and that of the capuchin monkey (*Sapajus apella*), we explored Felsenstein’s independent phylogenetic contrast approach (25) to measure the influence of phylogeny on microglial morphology.

To investigate microglia shape changes in a reactive state (non-homeostatic conditions) in small and large brains, we compared *M. musculus* (unpublished results) and *C. penicillata* (26) models of antibody-dependent enhancement of dengue disease, and reanalyzed the influences of an exacerbated inflammatory response following a peripheral virus infection on microglia morphological responses of these two species.

Finally, we re-evaluated our previous findings that explored the morphological response of microglia associated with sublethal encephalitis induced by RNA arbovirus in *M. musculu*s; we determined the influence of age and environmental enrichment on the neuroinvasion, virus clearance and damage to the CNS in relation to microglial morphology (15, 27).

## Morphological Diversity of Surveillant Microglia Across Species Under Homeostatic Conditions

Comparative morphological studies are now supported by molecular approaches, especially phylogenetics, which are increasing our understanding on morphophysiology and evolution of adaptive responses to environmental challenges (28).

The modern integrative biologist now faces the challenging task of understanding multiple physiological and genetic systems of the organisms they study, and there is an increasing need to look beyond established areas of expertise and drawn upon the experience of the wider scientific community (29). Unravelling microglia morphophysiology under homeostatic and non-homeostatic conditions is a good example of these new challenging times.

In all species investigated to date, microglia under homoeostatic conditions show delicate and ramified branches oriented radially from a small elliptical soma (2, 19). Microglia do not have a diversity of shapes, which can undergo back and forth morpho-functional changes, that they face to support neuronal and distinct immune functions under homeostatic and disrupted conditions (14, 19, 30–32).

Recently, it was demonstrated that the density of microglial cells in brains of different species and in the different structures of each individual brain are similar (7). These authors suggested that due to this constant density, microglia may have a similar average size in the evolution of mammals, with the same volume of tissue being monitored by each cell. However, others have shown that in a large number of species, microglia morphology varies across them with a conserved core program of orthologous genes, from rodents to humans (6).

Here, we examined microglia morphology in three species with similar body weights and brain volumes, i.e. the mouse (*Mus musculus*) as a terrestrial species, the lesser bulldog bat (*Noctilio albiventris*), and semipalmated sandpiper (*Calidris pusilla*) with aerial locomotion.

Although previous studies demonstrated that bats’ and birds’ have separate evolutionary origins, they share aerial mobility, smaller body weight and brain size, and differential adaptative phenotype responses for flying (33–36). In contrast to birds and bats, mice do not need to meet the weight and volume constrains imposed by flying (37, 38).

**Figure 1:**
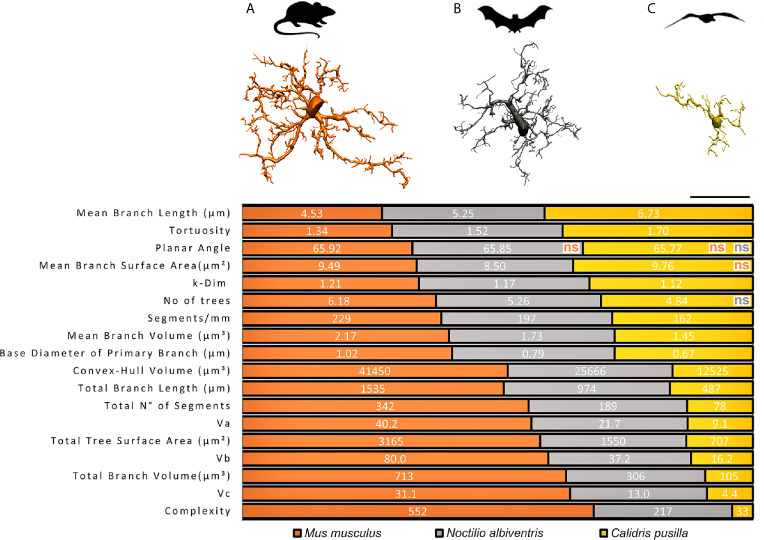
Graphical representation of the comparative analysis of morphometric features between species. Top, schematic illustration of *M. musculus* **(A)**, *N. albiventris* **(B)** *C. pusilla* **(C)** with correspondent three-dimensional reconstructions of microglia performed in the dentate gyrus of mammals and in the hippocampal V region of *C. pusilla*. 3D representative reconstructions of microglia are closest to the “mean cell” of each species. All morphometric features comparisons between species are significantly different (p < 0.05), except those indicated as non-significant (ns), and reproducing the color of the species being compared with *C. pusilla*. Three distinct mean values of each morphometric variable, one for each species, are exhibited on each line over three distinct colored areas as follows: orange, gray and yellow shaded areas corresponding to *M. musculus*, *N. albiventris* and *C. pusilla*, respectively. Note that the extent of each color shaded area on each line are the mean percentual values of each species. Scale bar = 25 µm.

Based on three-dimensional reconstructions of microglia immunolabeled with antibodies for ionized calcium binding adaptor molecule 1 (Iba1), as a marker for microglia and macrophages across species (39), a comparative analysis of the three-dimensional morphometric features of microglia of dentate gyrus of *C. pusilla* (24), *N. albiventris* (unpublished results) and *M. musculus* (23), with correspondent 3D microglia reconstructions of the cell closest to the “mean cell” of each species is shown in **Figure 1**. To choose the representative mean cell of each species, we used the distance matrix (distance between microglia pairs) to obtain the sum of the distances of each cell in relation to all the others. We assumed that the cell that best represents each group shows the least sum of distances. The matrices were constructed using all morphometric variables with the combination of all cells of a given group, followed by the weighted calculation of a scalar Euclidean distance between the cells. We used STATISTICA data analysis software system, version 12. StatSoft, Inc. (2014) to get the Euclidean distance matrices (distance between microglia pairs) and the sum of distances of each cell in relation to all the others (See **Table S1**for total number of cells in each species).

From *M. musculus* to *C. pusilla*, a progressive decrease in the number of branches is observed, with the microglia of *N. albiventris* occupying an intermediate position between the most branched microglia in *M. musculus* and the minimally branched in *C. pusilla* (**Figure 1**). Note that all, but few, of the comparisons of morphometric features in **Figure 1** were significantly different.

Morphological features with no statistical differences were only observed for Mean Branch Surface Area between *C. pusilla* and *M. musculus*, and for Number of Trees between *C. pusilla* and *N. albiventris*. It is important to highlight that, on average, the microglia of *M. musculus* survey a Convex Hull Volume of tissue that is almost twice that of *N. albiventris* and three times the one of *C. pusilla*. The *M. musculus* microglia exhibit on average of 2.5 and 16.7 fold greater Morphological Complexity than microglia of *N. albiventris* and *C. pusilla* respectively, and with a Number of segments that are on average 1.8 and 4.4 fold greater than for *N. albiventris* and for *C. pusilla*. These morphological differences may have important implications for the physiology of microglia across species of similar body weight and brain volumes. For example, the relative energetic cost per surveilled unit of tissue volume may not be the same across species, imposing a differential tradeoff between the contribution to neural physiology and the immune surveillance (40).

**Figure 2:**
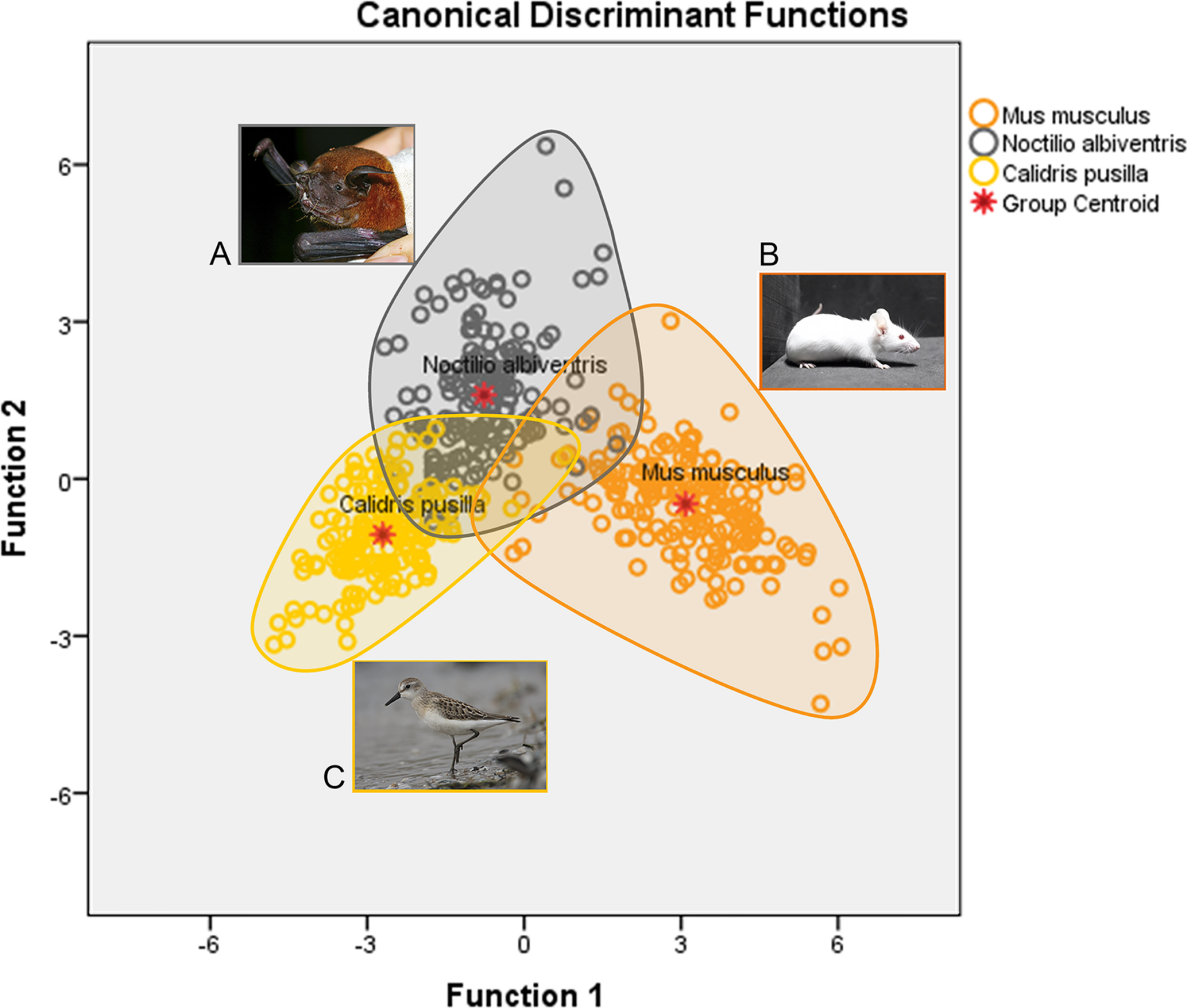
Graphic representation of the canonical discriminant function analysis of three-dimensional microglia reconstructions in the dentate gyrus of *N. albiventris* **(A)**, *M. musculus* **(B)** and hippocampal V region of *C. pusilla* **(C)**. Open color circles illustrate the microglia distribution in each species. The ellipsoid for *N. albiventris* (gray circles) intercepts both *M. musculus* (orange) and *C. pusilla* (yellow), with a greater overlap with *C. pusilla* Function 1, explains 82.0% of the variance (for details of statistical analysis see **Supplementary Material SM1**).

**Figure 2** is a graphic representation of canonical discriminant functions of all microglia morphological features of *M. musculus*, *N. albiventris* and *C. pusilla*. Three different ellipsoids, each one corresponding to a different species, occupy distinct regions of the Euclidian space. The ellipsoid representing the microglia of *N. albiventris* shows small, but asymmetric intersections with the ellipsoids of *C. pusilla* and *M. musculus*, sharing a greater region with the ellipsoid of *C. pusilla*. Thus, taking the *M. musculus* ellipsoid as a reference point to the other species, the greater Squared Mahalanobis Distance (which measures multivariate distances between the ellipsoids in arbitrary units) was found in *C. pusilla*, and the smaller one in *N. albiventris*. All distances between ellipsoids were statistically significant and the smaller distance occurred between *N. albiventris* and *C. pusilla*. For detailed statistical analysis see **Supplementary Material SM1**. Note that the morphology of microglia within each species is relatively homogeneous and this is reflected in cell spatial distribution concentrated within each ellipsoid (**Figure 2**). The separation between the ellipsoids of each species reflects the interspecific morphological differences. In fact, the microglia of *C. pusilla* monitors, on average, less tissue volume per microglial unit, and shows simpler trees, which facilitates the mobility of their branches and the penetration into areas of compact tissue. At the other extreme are those of the mouse, with more complex trees, which makes the penetration of their branches into regions of compact tissue more difficult. In contrast, as mouse microglial trees fill a larger volume, their capacity for monitoring by volume unit of brain tissue is greater. These differences may imply differential metabolic costs per unit of tissue sampled by every microglial cell of each species. The morphometric properties of the bat microglia seem to occupy an intermediate situation, despite exhibiting characteristics closer to the mouse microglial morphology.

## Phylogenetically Independent Contrast and Microglia Morphometric Features

To integrate the increasing availability of phylogenetic trees with comparative biology, Felsenstein (25) developed phylogenetic independent contrasts (PIC). The PIC approach provides a specific evolutionary scenario that allows researchers to conduct statistically robust regression analyses for trait comparisons using information for all species in the phylogenetic analysis, instead of only the sister species comparisons (25). Bird and mammal phylogenies have been successfully reconstructed using deoxyribonucleic acid (DNA) sequences of the subunit I of the mitochondrial enzyme of COI (41–43) and nuclear DNA sequences of RAG-1 (44–46). Because these genes show different rates of evolution, we used both data sets to improve resolving power and increasing the coverage of our analysis (47) considered tree topology and branch lengths in a phylogeny based on mitochondrial and nuclear genes. Once obtained, these parameters provide a set of independent contrast values between species that can be used in statistical regression and/or correlation procedures. A similar approach was successfully adopted in recent studies related to the morphological complexity of astrocytes in shorebirds (48).

To fulfill the requirements of Felsentein’s approach we added another distantly related species with a larger brain and arboreal locomotion, the capuchin monkey (*Sapajus apella)*, a New World monkey with remarkable cognitive capacities (49–53). The microglia morphology of semipalmated sandpiper (*C. pusilla*), mouse (*M. musculus*), lesser bulldog bat (*N. albiventris*) and capuchin monkey (*S. apella*), together with the DNA sequences of COI and RAG-1 genes of each species, were used to search for phylogenetic influences on microglia morphology across species.

DNA sequences for COI (54) and RAG-1 (55) were obtained from GeneBank (accession code in **SM1**) for all species. Sequences were aligned using ClustalW (56) implemented in BioEdit 7.2.5 (57) and concatenated using the program SequenceMatrix 1.8 (58). The software PartitionFinder (59) was used to choose the most appropriate model of nucleotide evolution for phylogenetic analysis according to Bayesian Information Criterion (BIC). We used the “Speciation: Yule Process” tree prior (60) implemented in Beast v1.7.5 software to estimate species trees with two independent runs of 5x10^8^ MCMC (Markov chain Monte Carlo). Sampling occurred at every 50,000 generations. The effective sample size (ESS) values were verified to determine the stationary posterior distributions of the parameters in Tracer 1.6 (61). Log files and trees were combined using the LogCombiner 1.7.5 application (62), while the Maximum Clade Credibility (MCC) tree was obtained from TreeAnnotator 1.7 (62) by applying a burn-in value of 10% of the total trees (1000). We calibrated two nodes in the species tree using the mammal time tree of Foley (63), setting a mean value of the most recent common ancestor (tmrca) of the mammals as 86.9 my, and a mean value of tmrca between Rodentia + Primates as 77.84 my. To obtain PIC values, we used the PDAP module (64) inside Mesquite Software Version 3.6 (65). The PIC analysis allowed us to test for phylogenetic influences on all 21 morphometric features of microglia three-dimensional reconstructions for all species.

**Figure 3:**
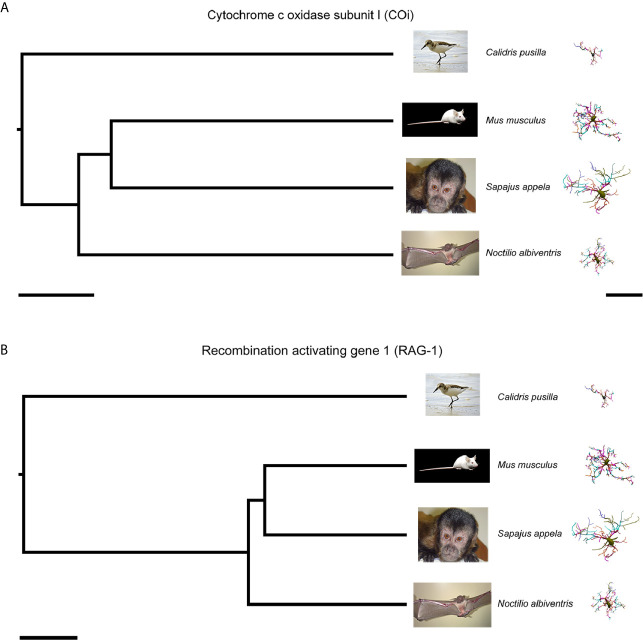
Phylogenetic trees based on the DNA sequences of the mitochondrial COi **(A)** and nuclear **(B)** genes for *Calidris pusilla*, *Sapajus apella*, *Mus musculus* and *Noctilio albiventris* with corresponding three-dimensional reconstructions of microglia. The representative cell is closest to the “average” cell for each species. To select them, the distance matrix was used to obtain the sum of the distances of each cell in relation to all the others. We assumed that the cell that best represents each group would have the least sum of distances. See **Table 2** for molecular markers used for phylogenies based on COi and RAG-1. COi, cytochrome oxidase subunit 1; RAG-1, Recombination activating gene 1. Scale bars for phylogeny A = 20Ma, B = 30Ma; scale bar for microglia reconstructions = 40 µm.

**Table 1** shows the median values of morphometric features used to estimate phylogenetically independent contrasts and corresponding p values for two tail t-tests of significant phylogenetic influence. We tested 21 morphometric variables and found that phylogenetic effects cannot explain morphological differences across species. Indeed, only two out of 21 morphometric features were influenced by phylogenetic differences based on mitochondrial COI and RAG-1 genes, namely the mean branch length for COI and the mean branch surface area for RAG-1. (See **Table 2** for molecular markers used for COI and RAG-1 phylogenies). The phylogenetic trees generated for COI and three-dimensional reconstructions of microglia closest to the “mean” cell of each species are shown on **Figure 3**.

**Table 1:** Median values used to perform the Phylogenetically Independent Contrasts and the p values for each gene.

Morphometric features	Median values				PIC 2-tailed p-value	
	*M. musculus*	*C. pusilla*	*S. apella*	*N. albiventris*	RAG1	COI
**Total Segment Length (µm)**	1494.500	451.200	574.600	887.550	0.886	0.136
**Average Segment Length (µm)**	4.292	6.144	5.719	5.216	0.989	0.028
**Average Tortuosity**	1.267	1.666	1.138	1.526	0.895	0.494
**Average Surface Area (µm²)**	8.933	8.984	9.945	8.442	0.035	0.827
**Total Branch Volume (µm³)**	686.883	93.611	187.445	286.827	0.780	0.212
**Average Branch Volume (µm³)**	2.026	1.303	1.698	1.580	0.504	0.817
**Base Diameter (µm)**	1.009	0.665	0.860	0.775	0.414	0.927
**Total Number of segments**	344.000	72.000	102.500	177.000	0.854	0.169
**Segments/mm**	233.005	162.704	174.905	191.671	0.882	0.100
**Number of trees**	6.000	4.000	6.000	5.000	0.735	0.704
**Surface (µm²)**	3101.710	636.142	1055.990	1392.895	0.752	0.232
**Complexity**	425034.000	21374.500	35488.200	150908.500	0.795	0.265
**Planar angle**	65.873	65.390	49.480	66.003	0.444	0.700
**Convex Hull Volume (µm³)**	38174.900	10479.100	10519.150	23164.550	0.911	0.160
**Convex Hull Surface (µm²)**	6989.170	2897.745	5396.140	4674.515	0.166	0.876
**Convex Hull Area (µm²)**	2392.000	909.438	2555.740	1492.625	0.917	0.577
**Convex Hull Perimeter (µm)**	187.450	120.900	196.850	150.050	0.880	0.631
**k-Dim**	1.217	1.115	1.088	1.172	0.776	0.335
**Total Vertex A**	40	8	14	20	0.838	0.135
**Total Vertex B**	77	14	22	33	0.757	0.258
**Total Vertex C**	31	4	8	12	0.745	0.257

Red values indicate that the variable was statistically significant in PIC analysis.

**Table 2:** Molecular markers used for Cytochrome *c* oxidase subunit 1 (COI) and Recombination activating gene 1 (RAG-1) phylogenies with GenBank/The Barcode of Life Database accession numbers.

Species	COI	RAG-1
*Mus musculus*	AB444046.1	AY011883.1
*Calidris pusilla*	AY666224.1	KC969130.1
*Sapajus apella*	KY173083.1	HM759116.1
*Noctilio albiventris*	MH886531.1	AF447509.1

Although our detailed 3D reconstructions of microglia showed distinct morphometries in distantly related species, these morphological differences could not be explained by phylogenetic influences. It is therefore relevant to examine other factors that may explain differential morphology between species (6, 7, 66–68). Here, we discuss the hypothesis that microglial morphological differences may be associated, at least in part, with different cognitive capacities.

## Microglia Morphology and Cognitive Performances

Microglia and their gene expression are key mediators of neuronal circuitry formation, function, and plasticity during normal physiological conditions (69). During the process of consolidating learning and memory, selective neuronal groups are recruited for learning, based on their level of excitation and synaptic activation, and this process is followed by subsequent strengthening of pre-existing synapses, formation of new connections and elimination of unnecessary ones (70). Shape, function and plasticity of the synapses underlying learning are especially interrelated in the hippocampus, a region whose integrity consolidation conditions episodic memory (71–73). As the hippocampus matures, lasting changes in the structure of synapses occur in association with long-term potentiation (LTP), replacing the intense synaptogenesis of the developmental period by enlarging and clustering mature brain synapses (74). These synaptic rearrangements are selective and strengthen the circuits related to the task being learned (75). In addition, functional magnetic resonance studies have shown that, comparing the activity of the hippocampal subfields to each other, the dentate gyrus (DG) is more active than Ammon’s horn (CA1-CA2-CA3) and subiculum, and that in both the coding process and information retrieval, the rostral DG is more active than caudal DG (76). When adult rats were trained to remember the spatial location of an object, synapse remodeling was observed 6 hours later in the molecular layer of the dorsal (septal) DG (DG-Mol) (77), and this remodeling was dependent on microglia (78, 79). Under homeostatic conditions microglia extend processes continuously and quickly, contacting synapses that are related to the ongoing experience, contributing to synaptic plasticity through the release of cytokines and growth factors (67, 80). In addition, coordinated regulation of gene expression leads to LTP, an essential mechanism underlying memory formation (81). In fact, the generation of LTP through the N-methyl-D-aspartate receptor (NMDAR) activates transcription factors that promote the expression of genes responsible for its maintenance (82). Indeed, it has been demonstrated that through enhanced expression of the Arc gene, a key regulator of synaptic plasticity, miR-34a regulates basal synaptic efficacy in the adult medial perforant path-evoked synaptic transmission in the DG (83).

Microglia also contribute to selective structural plasticity by eliminating synapses through phagocytic mechanisms, which are essential for maintaining normal cognitive activity (84). Indeed, previous reports have shown that the dynamics of microglial processes are regulated by sensory experience and neuronal activity (10, 22, 85); for review see (22, 86). Recent findings show a significant association between signaling in the pathways of the immune system regulated by microglia, synaptic strength, and related behavior (87). It has been demonstrated that the genetic removal of microglia-derived brain-derived neurotrophic factor (BDNF) in mice, or the selective deletion of microglia, decrease the synaptic expression of mature N-methyl D-Aspartate (NMDA) receptor subunit ionotropic glutamate receptors (GluN2B). In such condition there is an increase of the receptor subunit GluN2A, without affecting the density of neurons or synapses in the cortex and hippocampus (88). Dendritic spines (the post synaptic membrane of most excitatory synapses) have a crucial role in synaptic transmission and plasticity underlying cognitive processes (70, 75) and in BDNF signaling through functionally antagonistic receptors, such as the tropomyosin receptor kinase B (TrkB) and the p75^NTR^, over the lifetime, which are associated with proper synaptic plasticity, as well as the regulation of dendritic spine number and structure (89).

The morphological expression of the consolidation of learning and memory depends on microglia synaptic remodeling, with more significant changes in synaptic morphology in the target layers of the entorhinal-to-DG projection (77). Because form precedes function, we thought that microglia morphology could reflect their activity, since microglia participate in the process of synaptic rewiring, and because LTP induced plasticity underlying learning and memory in the mature hippocampus shows significant intertwining of form and function (74).

Here we re-evaluated previous findings on microglia morphology and its selective correlation with individual performance in spatial learning and memory tasks, in rostral DG. In a previous report (90), morphometric features of reconstructed microglia of the molecular layer of DG and of the lacunosum molecular layer of CA1 were tested for the correlation with the spatial learning and memory rate of the New World capuchin monkey (*Sapajus apella*), using the paired associate learning test of the Cambridge Neuropsychological Test Automated Battery – CANTAB (90). CANTAB tests have been successfully used before in other primate species (91–95), and in humans (96–101).

**Figure 4:**
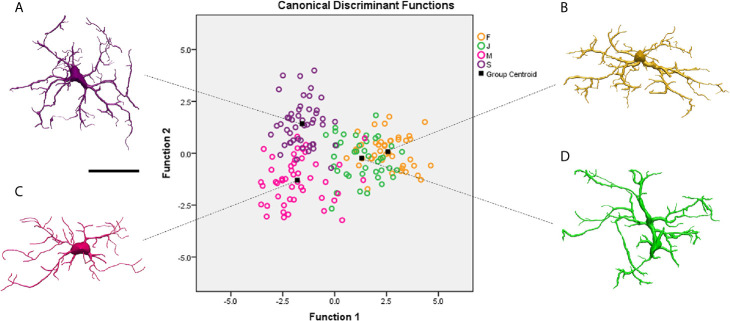
Graphical representation of the canonical discriminant function analysis of the morphometric characteristics of microglia obtained from three-dimensional reconstructions performed on the molecular layer of the rostral dentate gyrus of 4 individual capuchin monkeys (*Sapajus apella*): M, S, F and (J). Open circles of different colors illustrate the distribution of the microglia of each individual in the Euclidean space. Microglia of S **(A)** and M **(C)** with better learning rates occupy distinct regions in the left quadrants of the Euclidean space, while F **(B)** and J **(D)** individuals with lower learning rates share similar regions in the right quadrants of the Euclidean space. Function 1 and 2 explain 84.2% of variance (for statistical details see **Suplemmentary Material SM2**). Scale bar = 25 µm.

**Figure 4** shows discriminant analysis of microglia morphology of 4 different *Sapajus apella* individuals (S, M, F and J) with contrasting performances on the paired associates learning test (PAL) of the CANTAB battery (see **Supplementary Material SM2** for statistical details). Two subjects (M and S) completed the task after three and four training sessions respectively, and two others (J and F), only after 13 and 19 sessions, respectively. It is important to highlight, that while microglial clusters of fast learners (M and S) do not intercept with each other, slow learners (F and J) microglial clusters, showed a larger area of interception (**Figure 4**), suggesting that the correlation between morphological complexity and cognitive performance in paired associated learning task is not linear. See **Supplementary Material SM3** for correlation between the microglial morphological complexity (K-dim) and PAL task performances of slow (F and J) and fast learners (M and S) individuals.

Studies showed microglia from M subject has having the lowest morphological complexity mean values, but faster spatial learning and memory performance on the paired associates learning task (PAL) (90). The authors evidenced that performances in the PAL task and some morphological features of microglia of rostral region of DG-Mol, presented significant correlations, whereas none were found between performance and CA1 microglial morphology. It is reasonable to speculate that the formation of synapses in the learning and spatial memory network in *Sapajus apella* may be associated with lower microglia morphological complexity in the rostral DG. Because significant correlations between spatial learning and memory performance and microglia morphology were limited to the rostral region of DG, the results seem to be consistent with regional specializations of rostral and caudal regions of the monkey DG (102–105). These correlational differences between microglia structures and cognitive performance are an important observation and may suggest diverse physiological roles for microglia in the rostral and caudal DG of *Sapajus apella*. For detailed molecular mechanisms related to tissue-specific regulation of microglia form, function and dysfunction see (86, 106).

On the neuronal side it has been suggested that the learning process acts on microtubule dynamics at synaptic sites and these changes are critical for LTP and memory (107). It remains to be clarified, however, whether the learning process also requires dynamic alterations of microglia microtubules at synaptic sites of the molecular layer of rostral DG, and whether microtubule alterations lead to a reduction in microglia morphological complexity. To note that microtubules were shown to modulate the activity of Ras homologous (Rho) guanosine triphosphate (GTPases), which conversely are associated to microglia polarization (108). Recently, it was demonstrated that ablation of the small GTPase Rhoa in adult microglia led to spontaneous microglia activation and loss of synapses and neurons, leading to long-term synaptic plasticity impairment (109).

Thus, cytoskeleton reorganization determines changes in microglia morphology, and we may imagine that fast learners would have an increased number of microglia with lower mean values of morphological complexity than slow learners. Consistent with this view, the percentage of microglia with lower morphological complexity was asymmetrically distributed with a greater percentage in fast learner monkeys M (80.5%) and S (92.7%) than in slow learners monkeys F (31.4%) and J (66.7%) (90). Our findings in *Sapajus apella* demonstrated that within the same species and functional area, individual performance in spatial learning and memory tests were highly associated with differential morphology of microglia of the rostral DG, but not the CA1 lacunosum molecular layer, suggesting that microglia form and function in this species might be regulated in a tissue-specific fashion.

Taken together, our comparisons of the three-dimensional reconstructions of microglia of the DG of the capuchin monkey (*Sapajus apella*), mouse (*Mus musculus*), and hippocampal V area of semipalmated sandpiper (*Calidris pusilla*) with each other, and with microglia of DG of a bat (*Noctilio albiventris*), showed distinct morphologies across species. Using phylogenetic trees generated from DNA sequences of mitochondrial COI and RAG1 nuclear genes, Felsenstein’s phylogenetic independent contrast approach demonstrated that phylogenetic differences do not explain microglial morphological differences, suggesting the contribution of other factors such as differential cognitive performances across species.

## Microglia Response Under Arbovirus Attack and Metabolic Exchange Of Large and Small Brains

When the microglia react to an insult (acute or chronic), they change their functional and morphological phenotypes (2, 9, 16, 110). These responses seem to be region- (111, 112) and species-specific (6, 113), and are modulated by a variety of stimuli in health and disease (114–116). Virtually all brain diseases have their pathological progress influenced by microglial cells (2) that react them with multiple morphofunctional phenotypes (13, 116). Previous detailed reviews about microglia roles in distinct physiological and pathological contexts can be found elsewhere (8, 117–119) and are out of the scope of the present review.

It has been suggested that when natural selection differentially favors the brain or the immune system, increasing investment in one of those, the size or performance of the other will be reduced (120). When the immune response is activated by an infectious agent, the most obvious manifestation of the neuroimmune tradeoff is sickness behavior (121), a strategy for adaptive energy reallocation (122).

Owing to the tradeoff that exists between neural and immune systems, it has been predicted that larger brains would show less efficient immune responses compared to smaller brains, and that this evolutionary trade-off applies to the innate immune response, but not to the adaptive response one (40).

With this view in mind, we explored the differential response of microglia and neuroinvasion after dengue infection in *C. penicillata* and *M. musculus* as proxies for immune capabilities of small and larger brains.

Although most viral infections are concentrated in peripheral tissues (non-neurotropic viruses), many viral species reach the CNS where they can alter homeostasis and induce neurological dysfunction associated with life-threatening inflammatory diseases (123–126). Cross talk between neurons and microglia is, thus, essential for integrated actions, and small extracellular vesicles designated as exosomes have a crucial role in cell-to-cell communication, due to their content in lipids and proteins, but also in genetic material like miRNAs. There are a variety of miRNAs with key roles in microglia function and dysfunction, with aberrant expression of miRNAs being associated with microglia dysregulation and failure in sustaining CNS homeostasis; for review see (21).

During virus infections a sustained increase in extracellular adenosine triphosphate (ATP) induced by neuronal damage is sensed by microglia through the purinergic receptor P2Y12 followed by their activation and recruitment towards virally infected neurons, to exert phagocytic activity in minutes to hours (127). The number of P2Y12 receptors on the surface of microglia increases over twofold in response to viral infections (128). ATP is a strong chemotactic signal for young microglia, mediating rapid migration to the injury site (129). However, while young microglia respond to extracellular ATP by increasing their motility and becoming more ramified, aged microglia exhibits a contrary response, with low phagocytic ability (130), and becoming less dynamic and ramified (131). Thus, purinergic signaling is essential for the modulation of microglial movement in the normal and pathological brain (132, 133).

Facing homeostatic imbalance induced by any damaging agent (whether acute or chronic), changes in the morphology of microglia may be used as a proxy to judge about immune responses late in life, highlighting how important it is the investigation about the regulation mechanisms of microglia morphological changes (134). If we assume that form and function are intertwined, and that morphology precedes function both in developing (135) and mature brain circuits (136), we can examine any significant contribution from the morphometric analysis of microglia, either under homeostatic balance or imbalance (14, 17, 137); for recent review see (138).

In this regard, we re-examined our previous findings on the morphometric changes of microglia after a challenge with a non-neurotropic dengue arbovirus virus.

We explored the antibody-dependent enhancement (ADE) model of dengue infection (139, 140), which is associated with an enhanced CNS inflammatory response (141). We compared microglial morphological responses in a neotropical primate model, the black tufted marmoset (*Callithrix penicillata*) (26, 142), with that of a mouse model (unpublished results). The ADE of the host inflammatory response is a consequence of the presence of pre-existing anti-DENV antibodies, in a previously infected individual, binding and internalizing an infectious DENV viral particle of a different serotype in a subsequent infection (140, 143, 144). These subneutralizing antibodies from the primary infection cannot neutralize the virus. Instead, the antibody-virus complex binds to Fcγ receptors (FcγR) on circulating monocytes (140). These complexes of viruses and antibodies help the virus to infect monocytes more efficiently (145, 146). The result is an increase in the overall replication of the virus and higher risk of serious illness (139, 144).

**Figure 5:**
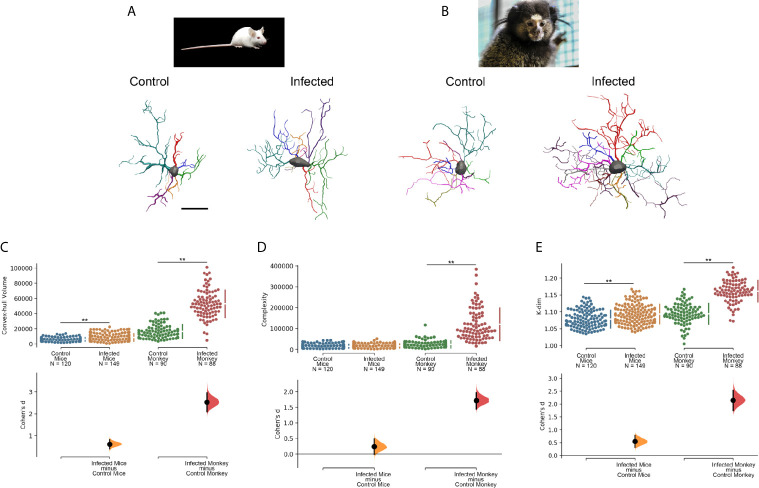
Microglia morphological responses to dengue infection in **(A)** mouse (*M. musculus*) and **(B)** black tufted marmoset (*C. penicillata*). **(C)** Convex hull volume, **(D)** Complexity and **(E)** K-dim with Cohen’s effect size that was calculated as the mean difference divided by the standard deviation of differences. 3D microglia reconstructions reflect the cell closer to the “mean cell” of each species. The representative (mean) distance matrix for each cell group was used in the calculation of the sum of each cell distances to all the others. We assumed the cell best representing each group by the least sum of distances. The matrices were constructed using all morphometric variables with the combination of all cells from a given group, followed by the weighted distance measure of a scalar Euclidean distance between the cells. Infected and Control designations over three-dimensional reconstructed cells indicate that microglia were reconstructed from dentate gyrus molecular layer of infected and control individuals, respectively. N indicates de total number of reconstructed microglia. Double asterisk (**) over connector bars denotes statistically significant difference p < 0.01. Scale bar = 15 µm.

**Figure 5** shows *C. penicillata* and *M. musculus* microglia three-dimensional reconstructions from control animals and individuals subjected to antibody enhancement dengue disease. The estimated overall effect size for Convex Hull Volume, Complexity and K-dim revealed that there were significant differential morphological responses of microglia in these models of enhanced inflammation. It should be noted that there are differences between Cohen’s effect size in the mouse and the black tufted marmoset, with 4–7-fold higher d values in the infected monkeys. Notably, no neuroinvasion was observed in the brains of the mice, whereas dengue virus antigens were detected in many areas of the brain of *C. penicillata*, suggesting that there is a more efficient immune response in mice. Likewise, after induced infection with dengue virus single serotype, viral antigens were detected in the cerebral cortex and peripheral organs of *C. penicillata* (142), while no neuroinvasion was detected in mice (147). It remains to be clarified whether this immunological efficiency is the consequence of an evolutionary tradeoff favoring the immune system homeostatic responses over the contribution to the preservation of neural homeostasis in *M. musculus* compared to *C. penicillata*.

As significant differences have been described between several gene ‘modules’ of the microglia of rodents and primates including phagocytic, complement and susceptibility genes to neurodegeneration (6), we suggest that the differential expressions of these gene modules may, at least in part, provide an explanation for the contrasting microglia morphological response observed in the distantly related mouse and black tufted marmoset. Further studies including transcriptomic analysis before and after antibody enhancement dengue infection will help to clarify this issue.

## Age and Sedentary Lifestyle Reduce Microglial Morphological Reactivity Under Neurotropic Arbovirus Infection

The movement of multiple microglial processes during their surveillance of the brain parenchyma surveillance is guided by the sensome (1, 148) that induces a variety of morphological identities with considerable crossover in function as they extend and retract processes (14).

The morphological parameters and process motility of microglia depend from several neuromodulators (149), and is increased by ionotropic glutamatergic neurotransmission and decreased by ionotropic GABAergic neurotransmission (150). Under homeostatic conditions constitutive bidirectional signaling between the neural and immune systems regulates immune cells, and vice-versa, in concert with the levels of neural and immune activity (151, 152).

We recently investigated the long-term influence of age and environment on the morphology of the microglia of the outer and middle thirds of the molecular layer of the DG of mice. We found that the enriched environment (EE) reduced the morphological diversity of the microglia of aged mice in such a way that a single morphotype was found in the aged mice (23), that it was associated with episodic-like memory preservation (153, 154).

**Figure 6:**
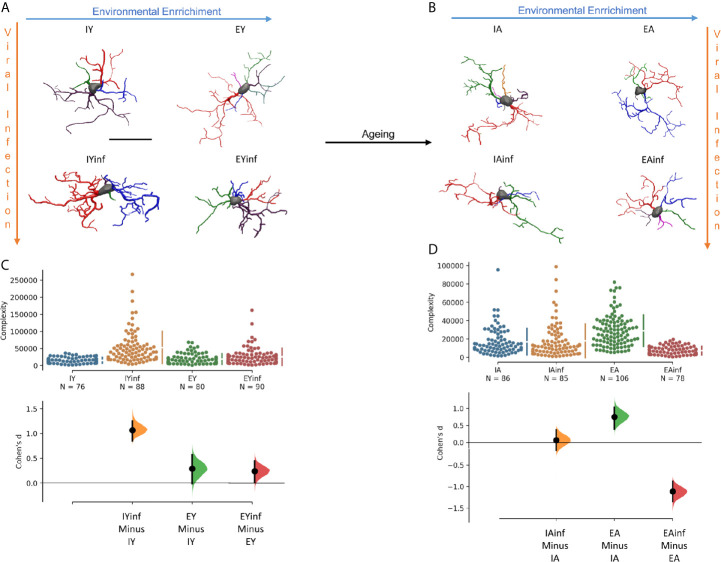
Age and environmental influences on microglial morphological response in a mouse model of sublethal encephalitis induced with Piry arbovirus. Three-dimensional reconstructions of the “mean cell” of each experimental group are shown, including control and infected young mice **(A)**, control and infected aged mice **(B)**. Microglia complexity of young **(C)** and aged **(D)** control and infected mice, under the influence of impoverished and enriched environments, with corresponding Cohen’s effect size was calculated as the mean difference divided by the standard deviation of differences. Notice that aging seems to minimize morphological changes, and effect size under neurotropic infection is greater in young mice maintained in impoverished environments. To choose the representative (mean) cell of each group, the distance matrix was used to obtain the sum of the distances of each cell in relation to all the others. We assumed that the cell that best represents each group is the one with the least sum of distances. The matrices were constructed using all morphometric variables with the combination of all cells of a given group, followed by the weighted calculation of a scalar Euclidean distance between the cells. IY, young mice raised in impoverished environment; EY, young mice raised in enriched environment; IA, aged mice raised in impoverished environment; EA, aged mice raised in enriched environment; IYinf, infected young mice raised in impoverished environment; EYinf, infected young mice raised in enriched environment; IAinf, infected aged mice raised in impoverished environment; EAinf, infected aged mice raised in enriched environment. Scale bar = 25 µm.

As environmental enrichment and physical exercise (155, 156), as well as aging (156–158), modulate microglia responsiveness, a key element in the pathological outcomes of arbovirus infections, we re-evaluated our findings related to the influence of age and environment on the microglial morphological response in a murine model of neurotropic virus infection (15). In **Figure 6**, we compared morphometric features of microglia in CA3 of control and infected young and aged mice when raised in contrasting environments mimicking sedentary and active lives, under Piry arbovirus sublethal encephalitis. We found evidence that under arbovirus attack, aging and sedentary-like lifestyle acting together significantly reduced microglial reactive response and associated with more severe and lasting behavioral changes.

**Figure 7:**
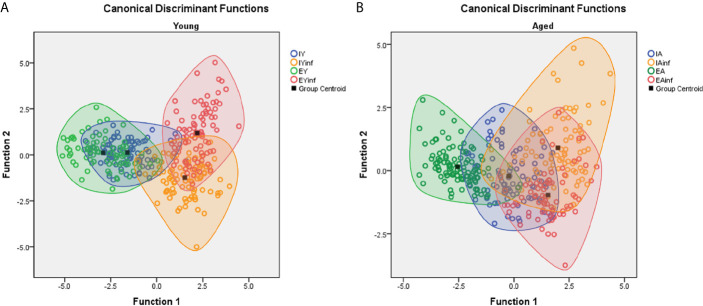
Discriminant function analysis to illustrate age and environmental influences on microglial morphological response in a mouse model of sublethal encephalitis induced with Piry arbovirus. Note that in young mice **(A)** there is a distinct distribution of microglia of infected (red and orange color open circles) in the right superior and inferior quadrants respectively, while in controls, the green and blue color open circles are in the central area of left quadrant. In contrast, the microglia of aged mice **(B)**, show larger areas of intersection between infected and control mice, indicating smaller influence of environment on microglia morphological response. Orange and red circles represent infected mice; Green and blue circles indicate control mice. IY, young mice raised in impoverished environment; EY, young mice raised in enriched environment; IA, aged mice raised in impoverished environment; EA, aged mice raised in enriched environment; IYinf, infected young mice raised in impoverished environment; EYinf, infected young mice raised in enriched environment; IAinf, infected aged mice raised in impoverished environment; EAinf, infected aged mice raised in enriched environment. Square black dot = ellipsoid center of each experimental group.

**Figure 7** shows discriminant function analysis applied to **Figure 6** data, where distinct distributions of microglia are noticed between infected (in the right superior and inferior quadrants) and control subjects (in the central area of left quadrants). Indeed, control mice, either from impoverished or enriched environments show many similar microglial morphometric features and, therefore, share large areas of their ellipsoids in the left quadrants. By contrast, infected mice kept in enriched and impoverished environments occupy the right superior and inferior quadrants, with less intersection of their ellipsoids, thus highlighting differential morphological microglia responsiveness.

However, when functional discriminant analysis is applied to the microglia of aged mice, they show larger areas of intersection in infected and control mice suggesting little differentiation of the microglial reactive response in mice maintained for life in either an enriched or an impoverished environment. See **Supplementary Materials SM4, SM5** for statistical details.

Due to the immunomodulatory influence of age and environment on microglia morphological response to virus neuroinvasion, it is important to examine the influence of these variables on the underlying mechanisms of immunological plasticity of the adaptive immune response.

Previous studies have shown that there is a significant beneficial influence of a healthy human life style on immune response in which regular moderate exercise and cognitive multidomain stimulation are key components (159–163) in the acquisition of the immune benefits (164); for recent reviews see (165, 166). Cognitive decline in aged rodents has received less investigation, and this seems to be associated with difficulties in isolating confounding, aged-associated changes in sensory and locomotor systems in behavioral tasks (167).

There is a consensus, however, that oxidative stress, DNA damage, mitochondrial dysfunction, excessive accumulation of misfolded proteins, damage to miRNA processing and inflammation are frequent causes of aging-related cellular dysfunctions (168), which may underly aging cognitive and immunological declines.

Modeling of human active lifestyles in mice and rats use environmentally enriched cages with increasing levels of novelty and complexity, to enhance somatosensory, visuospatial, cognitive and motor stimulation through voluntary exercise (169, 170). Running wheels, bridges, shelters, tunnels and toys are regularly moved inside the cage or replaced periodically, providing adequate stimuli to keep animals active, in order to achieve long term systemic beneficial effects, including higher immune responsiveness (171).

These animals, compared to sedentary animals raised in the impoverished environment of standard laboratory cages, exhibited differential immunological plasticity in both peripheral and central immune responses (172–175). In agreement, measurements interleukin-1β (IL-1β) and tumor necrosis factor-α (TNF-α) induction after novel object and exposure to accessories support the existence of an anti-inflammatory effect of environmental enrichment (176).

It has been also shown that short duration moderate exercise can improve survival in mice infected with a lethal dose of influenza virus (177). It has been shown that environmental enrichment and physical exercise, when combined, reduce brain cytokine expression (178), which may contribute to this outcome. Consistent with these findings, 6-month old mice under environmental enrichment, subjected to the neurotropic Piry arbovirus infection, increase T-cell infiltration and show less CNS cell infection by the virus and/or faster virus clearance, less microgliosis, and less damage to the extracellular matrix, than infected mice maintained in standard laboratory cages (27).

The signs of immunosenescence include low absolute numbers of T-cells, lower proportions of naive T-cells to differentiated effector memory T-cells, weak proliferative responses to mitogenic agents, and a ratio of CD4 to CD8 lymphocytes of less than 1.0 (179). Physical exercise and environmental enrichment can modulate immunity in aged humans (180, 181) and in experimental models (15, 27, 178, 182, 183). Indeed, it has been suggested that exercise might exert an anti-immunosenescence effect, delaying the onset of immunological ageing and rejuvenating aged immune profiles (179). In the hippocampal formation of aged mice, the response to several anti-inflammatory factors, including interleukin (IL)-4 and IL-13 is reduced relatively to adult mice, though exercise seems to have limited effects on modulating this response (183).

Our previous findings using Piry arbovirus neuroinvasion demonstrated an inverse association between age, behavioral impairments, and microglial host morphological changes (15). In fact, infected mice of advanced age, both in enriched environment (EE) and impoverished environments (IE), showed permanent reduction of burrowing activity and loss of olfactory discrimination, but only transient abnormalities in exploratory and locomotor activities. As burrowing activity requires hippocampal integrity and because the ventral hippocampus, DG, and septal regions were targeted by Piry virus, along with the olfactory nuclei and olfactory projections, we suggested inflammatory response and/or cell death along these pathways as probable causes (15). Microglial morphological changes in aged mice were less marked than those in young individuals, contributing to a reduced inflammatory response in immunosenescent individuals, and then more severe encephalitis outcomes and increased number of deaths (15). Like Piry virus infection, the olfactory pathways were also severely damaged during vesicular stomatitis virus (VSV) virus encephalitis induced by nostril inoculation, which affected similar anatomical targets within the anatomical olfactory pathways (184–187).

We can only speculate about mechsnisms that govern the underlying differential microglial 3D morphological changes under homeostatic and non-homeostatic conditions in young and in aged individuals? These three-dimensional morphological changes, which are strongly influenced by age and environment, are, of course underpinned by altered second messenger signaling. Gi-mediated decreases in cyclic adenosine monophosphate (cAMP) tone contribute to larger process extension, and Gs-coupled pathways induce process retraction (134). Though we have characterized the changes in the large processes of microglia, the extension of which is largely THIK-1 and P2Y12 dependent, microglial cells appear to conduct their surveillance of the homeostatic brain at the much smaller nanometer scale by means of tiny filopodia, which respond to stimuli by altering local concentrations of cAMP in a THIK-1 -independent manner; these local changes in cAMP, that are probably under the tight control of a-kinase anchor protein (AKAP), are also known to control the polarity of the microglial responses (134).

Morphological changes may associate with the time break window motility that include limited surveillance and enveloping sites of tissue damage controlled by P2Y12 activation and membrane potential (188). At the nanometric scale, filopodia also participate and depend on the local concentration of cAMP (134).

The microglia of mice from 18- to 20-month-old show a reduction in the number of ramifications and hypertrophy of the perinuclear cytoplasm supporting a change to a proinflammatory profile, as indicated by the elevated expression of inflammatory-response genes in transcriptomic analysis (189, 190). Between 3 months and two years of age, mouse microglia gets older, with significant numerical and morphological changes, which include increased density, increased soma area, and shorter and thicker processes impairing motility of their branches (106, 191, 192). Phenotypic variation in cellular senescence was recently indicated to involve four distinct stages: initiation (proliferation arrest), early (anti-inflammatory-state), full (increased inflammation and metabolism) and late senescence (decreased inflammation and metabolism), based on metabolism and senescence-associated secretory phenotype features. Such states may be influenced by diet, hormones, and epigenetics, and surely relate with the controversial findings on the role of chronic and increased neuroinflammation associated to aging and neurodegeneration (193).

It has recently been suggested that in the rat hippocampus a microglia-astrocyte interaction may induce microglial branching in the presence of inflammation and that the impairment of such interaction would be associated with inefficient microglia distribution, maladaptive morphology, and reduced clearance activity in aged rats (194).

Microglial morphology has been directly correlated to gene expression within the same brains using three-dimensional microscopic reconstruction and transcriptomic analysis (195). It has been shown that the total area and volume of Iba1 and P2Y12 immunolabeled microglial processes in CA3 of male and female mice subjected to lipopolysaccharide (LPS) challenge showed a significant inverse correlation with a robust index of microglia maturity, based on a ratio of selective up- and down-regulated genes, with much larger impact on both measures in males than in females (195).

It remains to be clarified, however, to what extent these correlations between gene expression and microglial morphology change under neurotropic virus challenge with the influence of age and environmental influence mimicking active and sedentary lifestyles.

## Non-Biological Sources of Variation

It is not uncommon to find contradictory results in comparative studies due to the use of different animal lineages, variations in histological procedures, different methods, or in the case of 3D reconstructions, dissimilarities in the stereological sampling approach to select cells for reconstruction and ambiguities in the definition of the objects and areas of interest (196). To reduce possible sources of non-biological variation in the present report, all samples of our previous findings were obtained with the same tissue processing protocols (perfusion, antigen retrieval, immunoreaction, dehydration, counterstaining, and clearing) and the specificity of the immunohistochemical pattern was confirmed using a control reaction that omitted the primary antibody (197–199). To obtain sufficient contrast between foreground and background we improved the signal/noise ratio with glucose-oxidase-DAB-Nickel peroxidase amplification method (200).

Microscopic 3D reconstructions are affected by mechanical factors due to vibratome sectioning and tissue dehydration procedures, inducing non-uniform shrinkage in the z-axis of the sections (201). Thus, estimates of modifications in the x/y dimensions during tissue processing cannot be linearly extrapolated to the z dimension across section. These methodological constraints impose limitations that must be considered when interpreting the results of all morphological studies based on 3D reconstructions. However, a reliable indication of severe shrinkage in the z-axis is the curling of branches, indicating that individual processes did not shrink at the same rate as the slice in which they were located. These effects tend to be of higher amplitude at the surface, decreasing in depth along the z-axis. To minimize this effect, we took our samples from the middle region of the z-axis, where the impact of these changes is expected to be minor (15). In addition, it has been demonstrated that in the z-axis (perpendicular to the cutting surface), sections shrink by approximately 75% of the cut thickness after dehydration and clearing (202). Based on those findings, all microglial reconstructions were corrected for Z-axis shrinkage, expecting that shrinkage would be 75% of the original value. No corrections were applied to X/Y axes, as it is expected that these dimensions do not change after histological dehydration and clearing. Moreover, we used the same software and hardware approaches for sampling, reconstruction, and analysis (Stereology, Neurolucida and Neuroexplorer, MicroBrightfield, Inc.), which guarantee systematic and random sampling selection of microglia across all regions of the areas of interest. Finally, to detect possible variations in the criteria for identifying and include only complete microglia arbors inside the area of interest, we underwent checking procedures of the results by having different investigators reconstructing microglia in the same regions using the same monoclonal IBA1 antibody as a marker for microglia and macrophages.

## Conclusion

Microglia morphological features under homeostatic conditions were found to diverge in the DG across species (*C. pusilla*, *N. albiventris*, *M. musculus* and *S. apella*) with large variation in tree branching pattern and cell size, not explained by phylogenetics differences. Other determinant factors for the differential microglia morphology across species may then include cognitive exercise, environmental stimuli, as well as aerial *vs.* terrestrial locomotion that may impose limits, regulating neuroimmune interactions. When microglia react to an insult (acute or chronic), they change their morphofunctional characteristics and phenotypic plasticity, with differential morphological responses in small and large brained species and these responses are influenced by age and environmental stimuli, with region and species-specific modulatory influences.

## Author Contributions

All authors contributed to the article and approved the submitted version. CP, DB, DA and DS participated in the data interpretation and writing of the final version.

## Funding

Fundação de Amparo à Pesquisa do Pará – FADESP/Pró-Reitoria de Pesquisa e Pós-Graduação da Universidade Federal do Pará – PROPESP Edital 2020-PIAPA; Coordenação de Aperfeiçoamento de Pessoal de Nível Superior – CAPES – Pró-Amazônia, Grant No. 3311/2013; Programa Ciências do Mar II; The Canadian Bureau for International Education (CBIE); Brazilian Research Council – CNPq Grant No. 307749/2004-5 and 471077/2007-0 for CP, Fundação Amazônia de Amparo a Estudos e Pesquisas do Pará – FAPESPA, ICAAF No. 039/2017. DB was supported by Fundação para a Ciência e a Tecnologia (PTDC/MED-NEU/31395/2017, LISBOA-01-0145-FEDER-031395 and UID/DTP/04138/2018-2021), as well as by Santa Casa da Misericórdia (ALS Research Grant). PV was supported by Brazilian National Research Council - CNPq grants No 573739/2008-0, 457664/2013-4 and 303999/2016-0.

## Conflict of Interest

The authors declare that the research was conducted in the absence of any commercial or financial relationships that could be construed as a potential conflict of interest.

## Supplementary Material

Supplementary Figure 1 Detailed statistical analysis of canonical discriminant function analysis of three-dimensional microglia morphological features in the dentate gyrus of *N. albiventris* (grey circles), *M. musculus* (orange circles) and hippocampal V region of *C. pusilla* (yellow). Function 1 explained 82% of the variance. Functions 1 and 2 are indicated on Structure Matrix Table.

Supplementary Figure 2 Detailed statistical analysis of canonical discriminant function analysis of three-dimensional microglia morphological reconstructions performed on the molecular layer of the rostral dentate gyrus of 4 individual capuchin monkeys (*Sapajus apella*), F (flo – orange circles), J (jac – green), M (mad – pink) and S (sme – purple). Microglia of S and M with better learning rates occupy distinct regions in the left quadrants, while F and J individuals with lower learning rates share similar regions in the right quadrants. Function 1 and 2 are indicated in the Structure Matrix Table and explain 84.2% of variance.

Supplementary Figure 3 Pearson’s correlation analysis between morphological complexity (K-Dim) of microglia of molecular layer of rostral region of dentate gyrus and PAL performances of slow (F and J) and fast (M and S) learner monkeys. Color circles and letters distinguish data from different individuals.

Supplementary Figure 4 Detailed statistical analysis of canonical discriminant function analysis of three-dimensional morphological reconstruction of microglia morphological response in young mice subjected to sublethal encephalitis induced with Piry arbovirus. Note a distinct distribution of microglia of infected (red and orange color open circles) in the right superior and inferior quadrants respectively, while in controls, the green and blue color open circles are in the central area of left quadrant. Function 1 to 3 are indicated in the Structure Matrix Table and Function 1 explain 83.0% of variance. Blue circle = IY, impoverished environment young control mice; Orange circle = IYinf, impoverished environment infected mice; Green circle = EY, enriched environment young control mice; Red circle = EYinf, enriched environment young infected mice; Square black dot = ellipsoid center of each experimental group.

Supplementary Figure 5 Detailed statistical analysis of canonical discriminant function analysis of three-dimensional morphological reconstruction of microglia morphological response in aged mice subjected to sublethal encephalitis induced with Piry arbovirus. The microglia of aged mice, show larger areas of intersection between infected and control mice, indicating less influence of the environment on microglia response of aged mice. Function 1 to 3 are indicated in the Structure Matrix Table and Function 1 explain 86.6% of variance. Blue and green circles represent microglia of control mice whereas red and orange represent microglia of aged infected mice. IA, impoverished environment aged control mice; IAinf, impoverished environment aged infected mice; EA, enriched environment aged control mice; EAinf, enriched environment aged infected mice; Square black dot = ellipsoid center of each experimental group.
