# Supplementary figures and images for: Microglial Morphology Across Distantly Related Species: Phylogenetic, Environmental and Age Influences on Microglia Reactivity and Surveillance States
Source: Front Immunol. 2021 Jun 18;12:683026. doi: 10.3389/fimmu.2021.683026 (PMC8250867; doi:10.3389/fimmu.2021.683026)

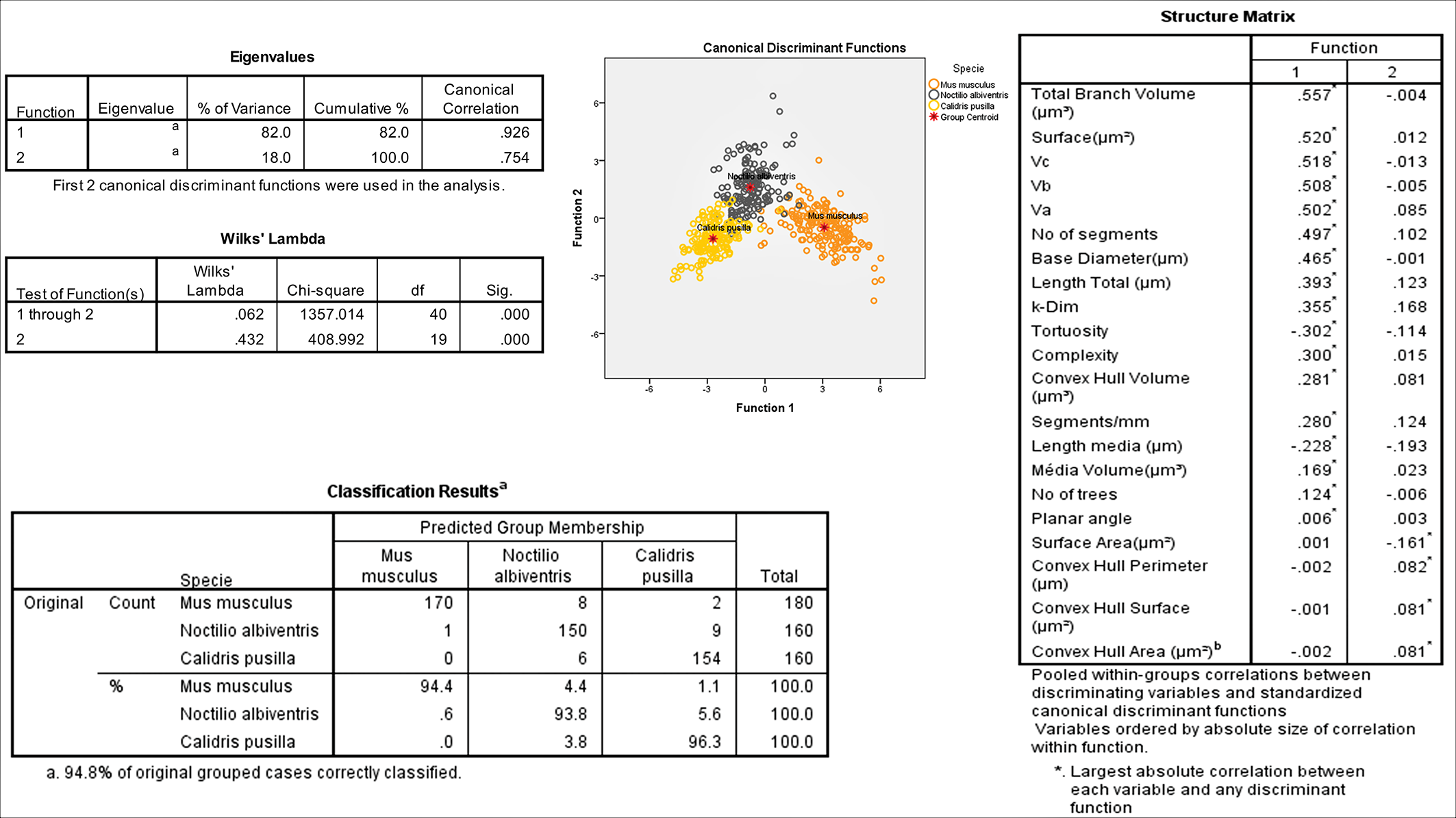

Supplement: Supplementary Figure 1 — Detailed statistical analysis of canonical discriminant function analysis of three-dimensional microglia morphological features in the dentate gyrus of N. albiventris (grey circles), M. musculus (orange circles) and hippocampal V region of C. pusilla (yellow). Function 1 explained 82% of the variance. Functions 1 and 2 are indicated on Structure Matrix Table. [file Image_1.tif]

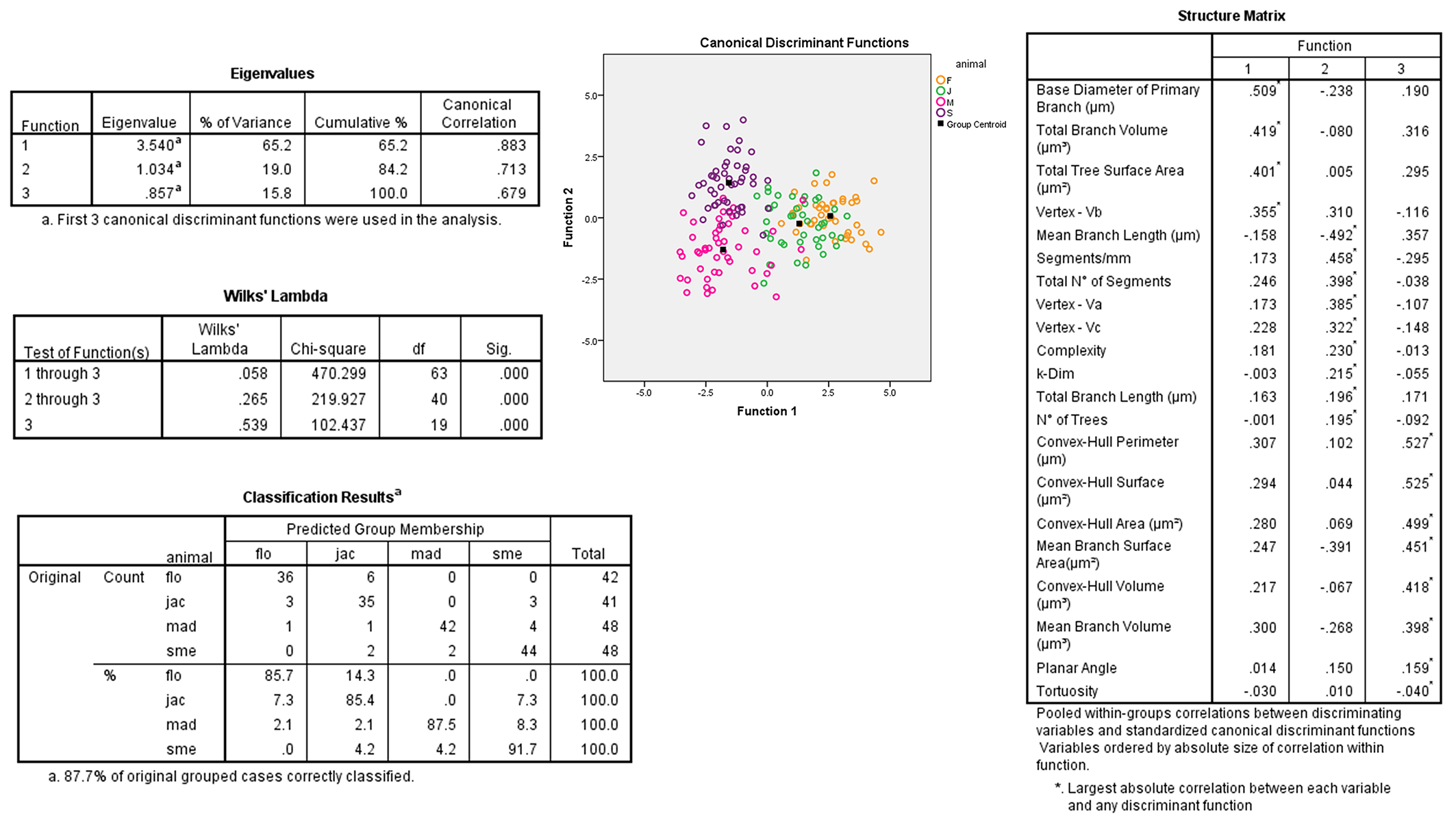

Supplement: Supplementary Figure 2 — Detailed statistical analysis of canonical discriminant function analysis of three-dimensional microglia morphological reconstructions performed on the molecular layer of the rostral dentate gyrus of 4 individual capuchin monkeys (Sapajus apella), F (flo – orange circles), J (jac – green), M (mad – pink) and S (sme – purple). Microglia of S and M with better learning rates occupy distinct regions in the left quadrants, while F and J individuals with lower learning rates share similar regions in the right quadrants. Function 1 and 2 are indicated in the Structure Matrix Table and explain 84.2% of variance. [file Image_2.tif]

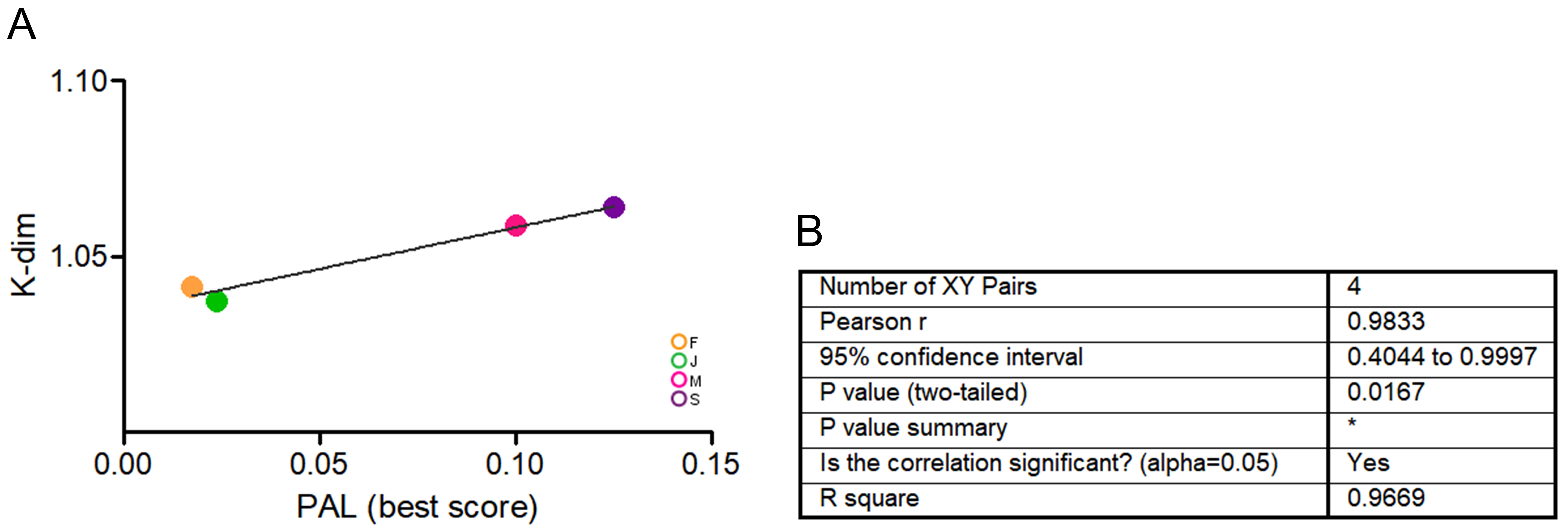

Supplement: Supplementary Figure 3 — Pearson’s correlation analysis between morphological complexity (K-Dim) of microglia of molecular layer of rostral region of dentate gyrus and PAL performances of slow (F and J) and fast (M and S) learner monkeys. Color circles and letters distinguish data from different individuals. [file Image_3.tif]

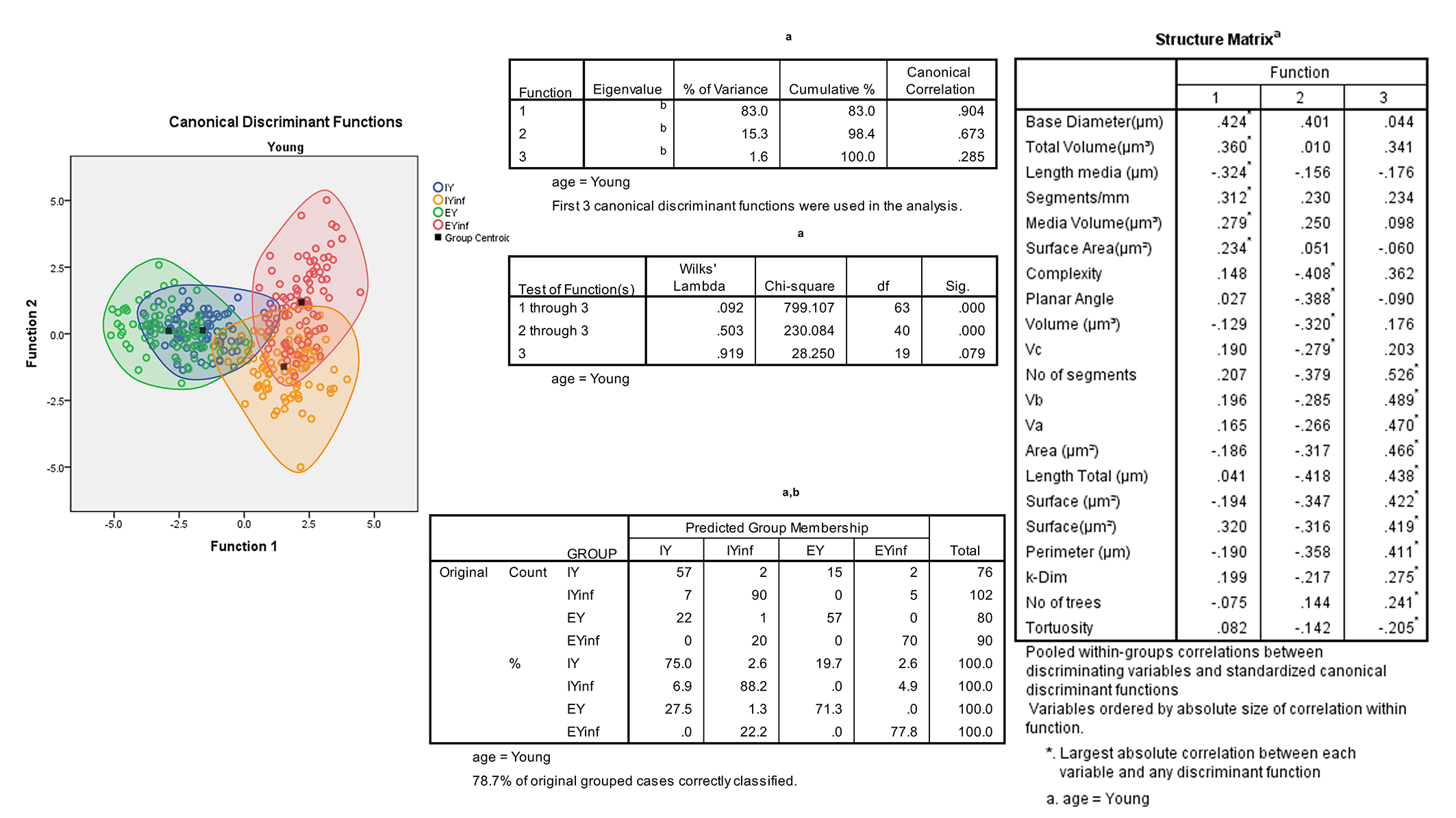

Supplement: Supplementary Figure 4 — Detailed statistical analysis of canonical discriminant function analysis of three-dimensional morphological reconstruction of microglia morphological response in young mice subjected to sublethal encephalitis induced with Piry arbovirus. Note a distinct distribution of microglia of infected (red and orange color open circles) in the right superior and inferior quadrants respectively, while in controls, the green and blue color open circles are in the central area of left quadrant. Function 1 to 3 are indicated in the Structure Matrix Table and Function 1 explain 83.0% of variance. Blue circle = IY, impoverished environment young control mice; Orange circle = IYinf, impoverished environment infected mice; Green circle = EY, enriched environment young control mice; Red circle = EYinf, enriched environment young infected mice; Square black dot = ellipsoid center of each experimental group. [file Image_4.tif]

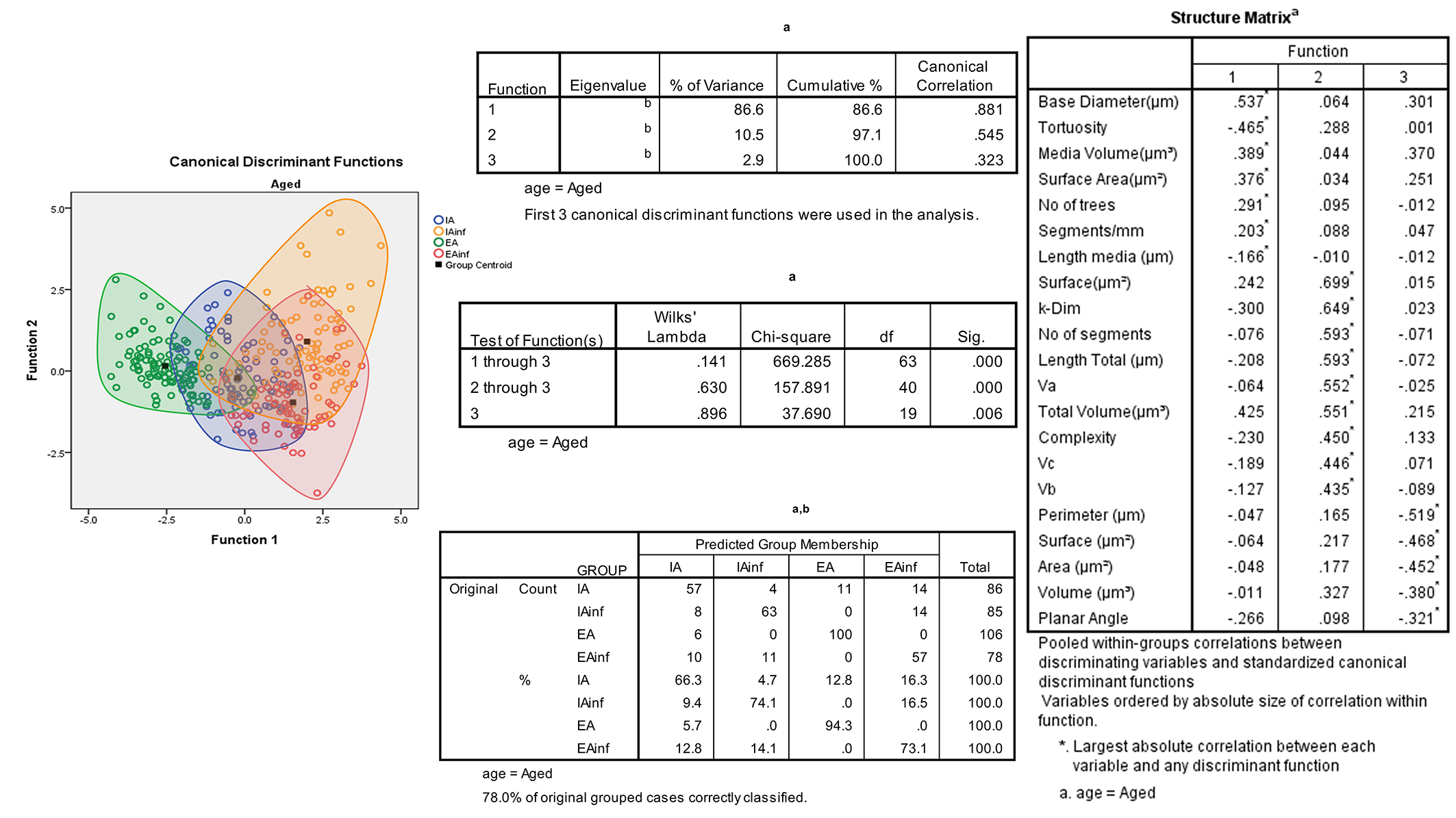

Supplement: Supplementary Figure 5 — Detailed statistical analysis of canonical discriminant function analysis of three-dimensional morphological reconstruction of microglia morphological response in aged mice subjected to sublethal encephalitis induced with Piry arbovirus. The microglia of aged mice, show larger areas of intersection between infected and control mice, indicating less influence of the environment on microglia response of aged mice. Function 1 to 3 are indicated in the Structure Matrix Table and Function 1 explain 86.6% of variance. Blue and green circles represent microglia of control mice whereas red and orange represent microglia of aged infected mice. IA, impoverished environment aged control mice; IAinf, impoverished environment aged infected mice; EA, enriched environment aged control mice; EAinf, enriched environment aged infected mice; Square black dot = ellipsoid center of each experimental group. [file Image_5.tif]
