# Supplementary material for: Microglial Morphology Across Distantly Related Species: Phylogenetic, Environmental and Age Influences on Microglia Reactivity and Surveillance States
Source: Front Immunol. 2021 Jun 18;12:683026. doi: 10.3389/fimmu.2021.683026 (PMC8250867; doi:10.3389/fimmu.2021.683026)
Supplement: Supplementary file 6 [file DataSheet_1.docx]

Table S1. Microglia from dentate gyrus of *N. albiventris, M. musculus and C. pusilla* used for the representative cell calculations of each species.

| Species | Number of individuals | Number of cells per individual | Total number of cells | Reference |
| --- | --- | --- | --- | --- |
| *Noctilio albiventris* | 4 | 40 | 154* | Unpublished data |
| *Mus musculus* | 3 | 60 | 180 | de Oliveira et al. (2020) and new data |
| *Calidris pusilla* | 4 | 40 | 160 | Diniz et al. (2016) and new data |

(*) a few outliers were removed based on standard deviation.
